# Effects of Pycnogenol® in people with post-COVID-19 condition (PYCNOVID): study protocol for a single-center, placebo controlled, quadruple-blind, randomized trial

**DOI:** 10.1186/s13063-024-08187-6

**Published:** 2024-06-15

**Authors:** Thomas Radtke, Lisa Künzi, Julia Kopp, Manuela Rasi, Julia Braun, Kyra D. Zens, Babette Winter, Alexia Anagnostopoulos, Milo A. Puhan, Jan S. Fehr

**Affiliations:** Epidemiology, Biostatistics and Prevention Institute (EBPI), University of Zurich (UZH), Zurich, Switzerland

**Keywords:** COVID-19, Post-COVID-19 condition, Pycnogenol®, Long COVID, SARS-CoV-2, Coronavirus

## Abstract

**Background:**

A significant proportion of the global population has been infected with severe acute respiratory syndrome coronavirus 2 (SARS-CoV-2) at some point since the onset of the pandemic. Although most individuals who develop coronavirus disease 2019 (COVID-19) recover without complications, about 6% have persistent symptoms, referred to as post-COVID-19 condition (PCC). Intervention studies investigating treatments that potentially alleviate PCC-related symptoms and thus aim to mitigate the global public health burden and healthcare costs linked to PCC are desperately needed. The PYCNOVID trial investigates the effects of Pycnogenol®, a French maritime pine bark extract with anti-inflammatory and antioxidative properties, versus placebo on patient-reported health status in people with PCC.

**Methods:**

This is a single-center, placebo-controlled, quadruple blind, randomized trial. We aim to randomly assign 150 individuals with PCC (1:1 ratio) to receive either 200 mg Pycnogenol® or placebo daily for 12 weeks. Randomization is stratified for duration of PCC symptoms (≤ 6 months versus > 6 months) and presence of symptomatic chronic disease(s). The primary endpoint is perceived health status at 12 weeks (EuroQol—Visual Analogue Scale) adjusted for baseline values and stratification factors. Secondary endpoints include change in self-reported PCC symptoms, health-related quality of life, symptoms of depression and anxiety, cognitive function, functional exercise capacity, physical activity measured with accelerometry, and blood biomarkers for endothelial health, inflammation, coagulation, platelet function, and oxidative stress. Investigators, study participants, outcome assessors, and data analysts are blinded regarding the intervention assignment. Individuals with PCC were involved in the design of this study.

**Discussion:**

This is the first trial to investigate the effects of Pycnogenol® versus placebo on patient-reported health status in people with PCC. Should the trial proof clinical effectiveness, Pycnogenol® may serve as a therapeutic approach to mitigate symptoms associated with PCC.

**Trial registration:**

The study is registered at ClinicalTrials.gov. :NCT05890534, June 6, 2023.

## Introduction

### Background and rationale {6a}

The coronavirus disease (COVID-19) pandemic has resulted in an ever-growing population of individuals recovering from a severe acute respiratory syndrome coronavirus 2 (SARS-CoV-2) infection. While most exhibit uncomplicated recoveries, a significant number experiences significant sequelae long after the acute illness has passed [1–7]. Population-based studies looking into PCC report a prevalence of about 20% of individuals with confirmed infection and about 6% of all infected with SARS-CoV-2 [8, 9]. While prolonged and persistent symptoms are more common among individuals who required hospitalization or experienced medical complications during the acute infection, data suggests that even some of those who experienced mild to moderate acute disease report persistent symptoms [2, 10]. Data from our population-based cohort study (Zurich SARS-CoV-2 Cohort) provide a comprehensive assessment of symptoms and information on the natural course of PCC [1, 3, 11]. In this cohort study, about 70% had mild symptoms, 20% had moderate symptoms, and 10% had severe symptoms and reported reduction in health status. Although symptom burden and severity of symptoms improved over time, about 18% of individuals with confirmed infection who were not vaccinated at the time of infection report persisting symptoms up to 2-years after infection [1].

With the increasing number of individuals affected from PCC there is also a growing effort from the scientific community to find effective therapeutic options [12]. Several countries have established a research agenda to prioritize research on PCC. Current research focuses on therapies to improve symptoms and functional status but also to understand pathomechanisms underlying post-COVID-19 sequelae (e.g., inflammatory processes, endothelial dysfunction) [13–16]. While the underlying pathophysiology of PCC remains unclear, some potential mechanisms include virus-specific pathophysiologic changes, ongoing immune stimulation from viral reservoirs in various tissues, persistent endotheliopathy, autoimmunity, hypercoagulation, and microthrombi as a result of an excessive inflammatory response [13, 14, 17].

It is important to find treatments with a favorable balance of benefits and harms. While a number of treatments under investigation may have considerable harms [12, 18, 19], Pycnogenol® may be an attractive treatment option since it is on the market for a long time with few side effects reported to date [20, 21] and some promising results in patients with PCC [20]. Pycnogenol® is a French maritime pine bark extract and primarily consists of procyanidins and their individual components, namely catechin and epicatechin, alongside phenolic acids [22]. It exerts antioxidative, anti-inflammatory, and antiproliferative properties and has been shown to improve vascular endothelial function, most likely due to an upregulation of endothelial nitric oxide synthase activity [21–25]. In people recovering from COVID-19, daily intake of 150 mg Pycnogenol® over a period of 12 weeks improved endothelial function and inflammatory and antioxidative biomarkers compared to control (usual treatment) [20]. Considering the potential beneficial effects of Pycnogenol® on disease characteristics of COVID-19 and PCC, it may improve the health status and alleviate symptoms in people with PCC.

### Objectives {7}

The primary objective of this randomized controlled trial (RCT) is to investigate the effects of Pycnogenol® versus placebo on self-reported health status (primary outcome) in people with PCC.

Secondary objectives are to assess the efficacy of Pycnogenol® versus placebo on PCC-related symptoms (e.g., fatigue, post-exertional malaise, dyspnea, cognitive function), health-related quality of life, symptoms of depression and anxiety, functional exercise capacity, physical activity, and biomarkers for inflammation, oxidative stress, coagulation, and endothelial function.

### Trial design {8}

This is a 12-week, single-center, placebo-controlled, quadruple-blind, parallel design superiority trial investigating effects of a daily dose of 200 mg Pycnogenol® versus a placebo. Randomization (1:1 allocation ratio) will be stratified for duration of PCC-related symptoms (i.e., ≤ 6 months vs. > 6 months) and presence of symptomatic chronic conditions.

## Methods: participants, interventions and outcomes

### Study setting {9}

This single-center study will be caried out at the Epidemiology, Biostatistics and Prevention Institute (EBPI) at the University of Zurich, Switzerland.

### Eligibility criteria {10}

#### Inclusion criteria

- Age 18 years and older
- SARS-CoV-2 infection confirmed by positive polymerase chain reaction (PCR) or rapid antigen test for professional use and/or physician confirmed diagnosis of PCC
- PCC (World Health Organization Definition [26]), i.e., ≥ 3 months after SARS-CoV-2 infection and ongoing or recurring symptoms for at least 2 months that cannot be explained by alternative diagnosis
- At least one of the following symptoms must be present at screening: fatigue, cognitive impairment (“brain fog”), dyspnea, post-exertional malaise
- Signed informed consent
- Sufficient language and cognitive skills
- Ability to participate in study visits at the study center or at home. Home visits are only possible if the travel distance by car is no longer than 90 min (one way) from the study center at the University of Zurich
- No foreseeable change in medication

#### Exclusion criteria

- Severe comorbidities such as liver or renal failure, advanced chronic obstructive pulmonary disease or pulmonary fibrosis requiring > 5 L oxygen/min at rest, active malignancy, advanced heart failure, and cardio-vascular events within the previous 24 weeks
- Acute respiratory or other infections
- Clinical diagnosis of psychiatric disease that is untreated and/or unstable
- COVID-19 vaccination less than 4 weeks prior to the baseline visit or during study participation
- Known intolerance to Pycnogenol®
- Regular intake of Pycnogenol®
- Being enrolled in another interventional study that may interfere with the current study

### Who will take informed consent? {26a}

Prior to screening, potential participants are provided with information about the study including its nature, purpose, expected duration, and potential risks and benefits. The principal investigator (PI), or their formally trained designees, explain these details to each potential participant. To ensure that participants have sufficient time to consider their participation, they are given at least 24 h between the first contact and the screening visit. Before any investigation takes place, the participant’s formal consent is obtained using the informed consent form. Participants are required to read, understand, and voluntarily agree to the participation in the study before signing and dating the informed consent form. They are given a copy of their signed document for their records.

### Additional consent provisions for collection and use of participant data and biological specimens {26b}

An additional consent form for further use of data and biological material is given to the participants. The additional consent is not compulsory for participation in the study.

## Interventions

### Explanation for the choice of comparators {6b}

We choose a patient-reported outcome as primary endpoint in this study (EQ-VAS) [27, 28]. To differentiate the potential effects of Pycnogenol® from the placebo effect, this study utilizes a placebo that shares identical physical characteristics and dosing regimen with the verum. Using a placebo is ethically justified, as there is no approved treatment for PCC, and no treatment of proved effectiveness will be withheld. Additionally, participants are not required to discontinue any PCC-related treatment or medication prior to enrolment into the study, except regular intake of Pycnogenol®.

### Intervention description {11a}

Table 1 provides an overview of study procedures including interventions. Following assessment of inclusion and exclusion criteria, eligible subjects are randomly allocated to the verum or placebo group.

**Table 1:**
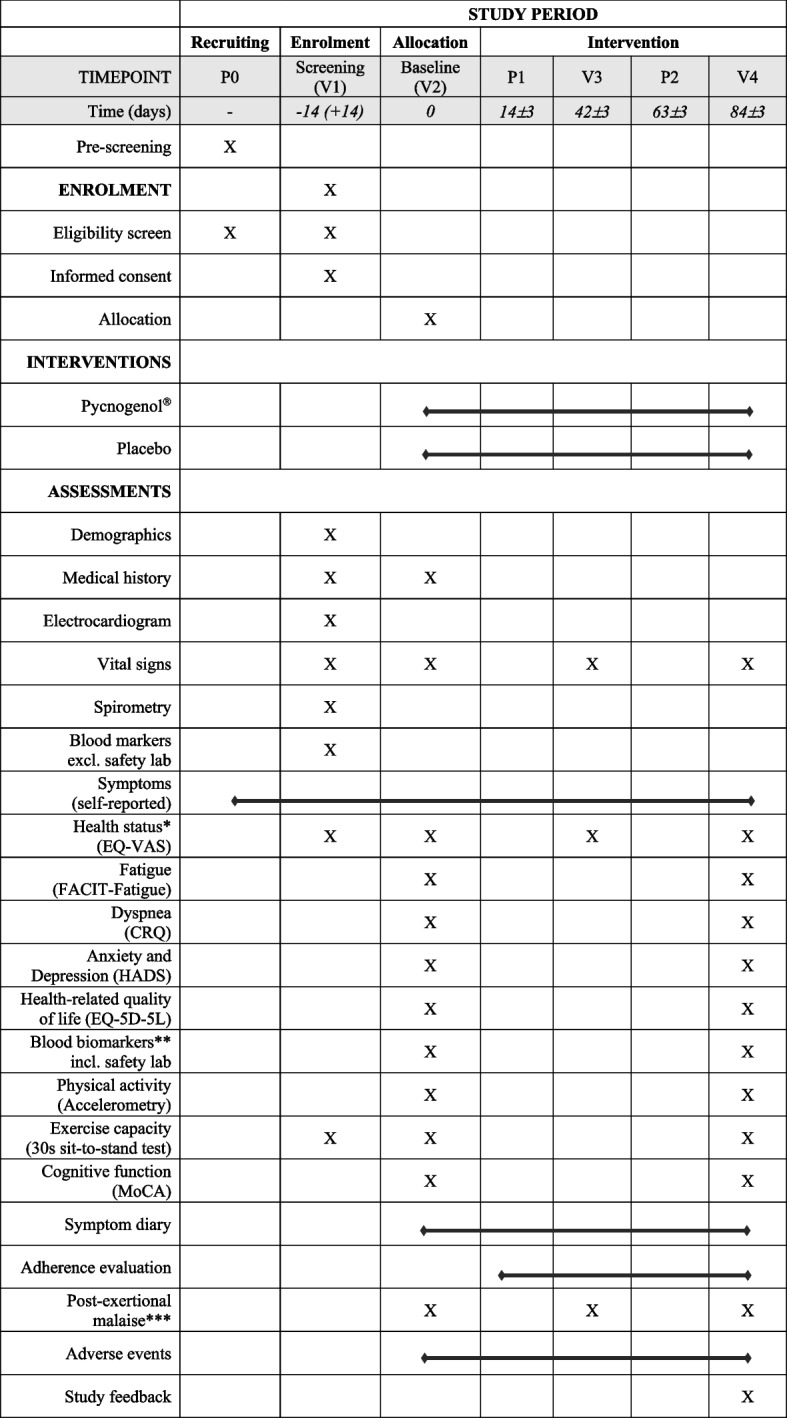
Overview of enrolment, assessments and outcome measures according to Standard Protocol Items: Recommendations for Interventional Trials (SPIRIT)

*Abbreviations*: *CRQ*, Chronic Respiratory Questionnaire; *EQ-5D-5L*, EuroQol Questionnaire (5 levels); *EQ-VAS*, EuroQol Visual Analogue Scale; *FACIT-Fatigue*, Functional Assessment of Chronic Illness Therapy—Fatigue Scale; *HADS*, Hospital, Anxiety and Depression Scale; *MoCA*, Montreal Cognitive Assessment Test

^*^Primary outcome: The EQ-Visual Analogue Scale is assessed daily over 7 consecutive days prior to the baseline visit (V2) and follow-up visit 2 (V4)

^**^Blood biomarkers of inflammation, coagulation and platelet function, oxidative stress, and endothelial function

^***^Study visits can potentially impose a burden on participants. At each visit, starting with the baseline visit (V2), participants are asked whether they experienced a worsening of their PCC-related symptoms within 3 days after the last study visit

^****^Upon completion of the study, participants receive a link to an online survey to request feedback regarding the study organization

Participants in the intervention group receive 200 mg Pycnogenol® per day for 12 weeks, with two capsules containing each 50 mg in the morning and two capsules in the evening. Pycnogenol® is a proprietary French maritime pine bark extract (Pinus pinaster subsp., atlantica, Pinaceae) produced by Horphag Research, Geneva, Switzerland. Pycnogenol® is standardized to contain 70 ± 5% procyanidins in compliance with the United States Pharmacopeia. Its compounds and gut metabolites are known among other actions for their antioxidant properties, anti-inflammatory activities, and stimulation of nitric oxide production in vascular endothelial cells [22].

Participants in the control group receive a placebo. The placebo capsules are manufactured in the same facility and have the same physical appearance, consistency, and weight as the verum. Like the participants in the verum group, participants in the control group take two capsules twice daily with or right after a meal.

### Criteria for discontinuing or modifying allocated interventions {11b}

In general, Pycnogenol® is well tolerated [20, 21, 29]. In this study, Pycnogenol® is supplied at a dose of 200 mg per day, with two capsules containing 50 mg in the morning and two capsules in the evening taken together or right after a meal to avoid potential gastrointestinal discomfort. Gastrointestinal discomfort is the most frequently reported treatment-related side effect in previous clinical trials [29]. Further reported treatment-related adverse events are dizziness, headache, and nausea [29]. All side effects, possibly related to the study product, will be recorded as adverse events. Adjustments in study product dosage or intake are not planned.

Upon withdrawal or exclusion, early termination will be recorded in the database, and the participant will be invited for a termination visit. If the participant is not able or willing to participate in a termination visit, a phone call will be offered instead. Moreover, we will ask the participant if they are willing to complete the study specific questionnaires to keep missing data to a minimum. The participant will be asked to return leftover study products to the study center.

### Strategies to improve adherence to interventions {11c}

**Fig. 1:**
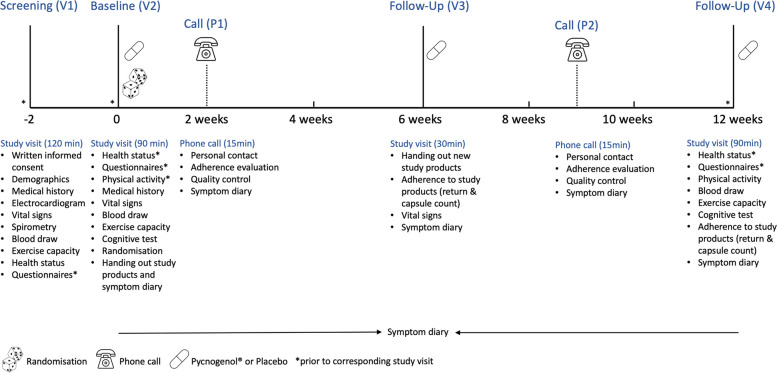
Participant timeline

To enhance the validity of our data and adherence to study products, we planned a short study visit six weeks and two phone calls two respectively 9 weeks after randomization (Fig. 1) to stay in close contact with study participants. During the phone calls, the importance of adhering to the study products and monitoring symptoms in the diary is discussed. Study products returned at the follow-up visits (V3, V4, Fig. 1) are counted and recorded in the database. In addition to the intention-to-treat analysis (primary analysis), we plan a per protocol analysis based on adherence to study products and investigate its potential impact on primary and secondary outcomes.

### Relevant concomitant care permitted or prohibited during the trial {11d}

Concomitant medication including herbal medications or supplements is principally allowed. Investigators instruct participants to keep their therapy regimen unchanged during the study period. This also applies for concomitant non-pharmaceutical PCC-related therapies. Participants are instructed not to start any new therapy, new preventive measures such as COVID-19 vaccinations, or intervention during the trial that may interfere with study outcomes. Those who plan to get vaccinated against SARS-CoV-2 will get the opportunity to get vaccinated at our center prior to the baseline visit.

### Provisions for post-trial care {30}

Participants terminating the study (either regularly or prematurely) with reported ongoing serious adverse events (SAE) or any ongoing adverse events regarding laboratory values or vital signs being beyond the alert limit will be asked to return for a follow-up investigation. This visit will take place up to 30 days after termination of the treatment period.

### Outcomes {12}

Table 1 provides an overview of assessments including primary and secondary outcomes.

#### Primary outcome

The primary outcome is self-reported health status assessed with the EQ-VAS, also known as the “Feeling Thermometer” [27, 28]. The EQ-VAS is numbered from 0 to 100 with 0 representing the “worst imaginable health” and 100 representing the “best imaginable health.” The scale is frequently used in people with chronic respiratory conditions [30–32] and has a well-established minimal important difference (MID) of 5–8 units [31]. In our trial, EQ-VAS is assessed daily over seven consecutive days prior to the baseline visit (V2) and again prior to the last study visit (V4). Mean EQ-VAS scores (i.e., pre- and post-intervention) will be computed, including at least 4 days. Post-intervention, EQ-VAS scores will be compared between the intervention and placebo groups and adjusted for baseline values. In addition, self-reported health status is assessed at screening (V1) visit. These data will be used for descriptive purposes. A minimal important difference (MID) of 8 units will be used for interpretation of the magnitude and clinical relevance of between-group differences in post-intervention EQ-VAS values [31].

#### Secondary outcomes

All secondary outcomes are assessed at baseline (V2) and after 12 weeks (V4), except post COVID-19 related symptoms. Symptoms are assessed using online questionnaires prior to study visits (V1, V2, V4) and a symptom diary that is completed on a weekly basis between the baseline and 12-week visit.

*Post COVID-19 symptoms* are assessed using self-reported surveys prior to the screening visit (V1), baseline visit (V2), and end of intervention (V4) [1, 3, 11]. Participants complete a symptom diary on a weekly basis including severity grading.

*Fatigue* is assessed using the 13-item Functional Assessment of Chronic Illness Therapy—Fatigue Scale (FACIT-Fatigue) [33]. A cut-off score of < 34 will be used for descriptive analysis, and norm values from a German population [34] will be used for comparisons. The scale is responsive to treatment, and triangulated MIDs have been established for various patient populations [35]. In a small study (*n* = 32) with people attending COVID-19 rehabilitation, the MID for the FACIT-Fatigue scale has been estimated between 2.7 and 3.6 points [36]. This estimate will be used for the interpretation of between-group differences in this study.

*Dyspnea* is assessed using the Chronic Respiratory Questionnaire (CRQ) dyspnea domain [37–39]. The CRQ dyspnea domain consists of five questions rated on a 7-point scale. The instrument is valid, reliable, and responsive, with a reported MID of 0.5 units [40], which will be used to interpret between-group differences.

*Cognitive function* is assessed by trained personnel using the German version of the Montreal Cognitive Assessment (MoCA) test. The MoCA test is commonly used to evaluate cognitive function in people with COVID-19 and PCC [41–44], with a cut-off score < 26 for impairment [42, 45]. The standard correction for years of education will be applied.

*Anxiety and depression* are assessed with the Hospital Anxiety and Depression Scale (HADS) [46–48]. The scale includes 14 questions with a 4-point Likert-type scale. It is a valid and reliable instrument with a triangulated MID of 1.5–1.7 units change [30, 49, 50]. A cut-off value of 7 or higher will be used to report the number of participants with depression and/or anxiety [51].

*Health-related quality of life* is assessed with the EuroQol Questionnaire (EQ-5D-5L) questionnaire [27, 28]. The questionnaire includes five dimensions scored on five levels of severity. The Dutch value set will be used to calculate EQ-5D-5L index scores.

*Functional exercise capacity* is assessed with the 30-s sit-to-stand (STS) test [52–54]. A familiarization test is done at the screening visit (V1). Heart rate, oxygen saturation, and ratings of perceived exertion and dyspnea are recorded before and after the exercise test. A between-group difference of ≥ 2 repetitions post-intervention will be considered clinically relevant [53].

*Physical activity* is measured with a triaxial accelerometer (ActiGraph wGT3X-BT, Pensacola, FL, USA) worn around the hip for 8 consecutive days before baseline (V2) and study end (V4). Data will be used for the analysis if at least 4 days (including a weekend day) of measurements with a minimum of 10 h wear time per day are recorded. Physical activity will be expressed as daily step count and time spent in different intensity domains using validated cut-offs [55–57].

*Biomarkers of endothelial health* include soluble thrombomodulin, von Willebrand Factor antigen, syndecan-1, and sVCAM-1 [58–61] and are measured using commercially available enzyme-linked immunosorbent (ELISA) or Luminex (bead-based immunoassay) assays following the manufacturers instruction.

*Biomarkers of inflammation* include C-reactive protein (CRP), interleukin-6 (IL-6), and leukocyte levels [60, 62]. Blood levels of CRP- and IL-6, as well as leucocyte numbers as part of the hemogram, will be determined for each participant.

*Biomarkers of coagulation and platelet function* include soluble CD40 ligand (sCD40L) and plasma soluble P-selectin (sP-selectin) [58, 63–66] and will be measured using commercially available ELISA or Luminex assays following the manufacturers instruction. In addition, D-dimers, international normalized ratio (INR), activated partial thromboplastin time (aPTT), and platelet concentration as part of the hemogram will be assessed.

*Biomarker of oxidative stress* will be the total antioxidant capacity (TAC) of blood plasma using the fluorescence recovery after photobleaching (FRAP) method.

*Biomarkers of kidney and liver function* include serum creatinine levels to assess estimated glomerular filtration rate (eGFR), aspartate aminotransferase (ASAT), alanine aminotransferase (ALAT), and gamma-glutamyltransferase (γ-GT).

### Safety outcomes

At each study visit (Table 1), vital signs (heart rate, oxygen saturation, blood pressure, body temperature) are measured. Blood pressure is measured in duplicate after resting for 5 min in sitting position (legs uncrossed) with a portable blood pressure monitor (Omron M3 Comfort). The average blood pressure value will be recorded and used for the descriptive analysis. Heart rate and oxygen saturation are measured in sitting position with a portable pulse oximeter (Beurer PO 80).

At the screening visit (V1), a 12-channel resting ECG (ELI™ 280, Hechingen, Germany) is performed with the participant in supine position (after at least 5 min rest) to screen for unstable cardiac disease. Furthermore, a blood sample is drawn to screen for other underlying causes for symptoms similar as those seen in individuals with PCC. The tests include blood count, ferritin, CRP, vitamin B12 levels, and thyroid-stimulating hormone (TSH) as well as kidney function (eGFR) and liver enzyme activity (ALAT, ASAT, γ-GT). Clinically relevant abnormal diagnostic laboratory parameters will be passed on to the participants so that necessary measures can be taken such as further diagnostics or treatment delivered through the participant’s attending physician.

### Other outcomes

*Adherence to the study products* is evaluated during the follow-up visits (V3 and V4) and the two follow-up phone calls (P1 and P2) using a standardized assessment form. During the first follow-up visit (V3), the number of capsules returned by the participant is counted and recorded in the database. New capsules are handed out to study participants. During the second follow-up visit (V4), the remaining capsules returned by the participants are counted and recorded in the database.

### Evaluation of perceived study burden and study organization

Post-exertional malaise is a frequent symptom in PCC, and we are aware that the study visits may pose a burden on the participants. At each visit, starting with the baseline visit, participants are asked whether they experienced a worsening of their symptoms within 3 days after the last study visit. Upon completion of the study, participants receive an invitation to an online survey to provide feedback regarding the overall study organization (e.g., duration of visits, breaks, number of assessments). These assessments aim to gather insights from participants on how to improve the design and organization of future trials for both this vulnerable patient population and others.

### Spirometry

In addition, spirometry is performed according to the standards of the American Thoracic Society and European Respiratory Society [67] using portable spirometry (PADSY Spiro, Spirosound, Medset Medizintechnik GmbH, Hamburg, Germany). Forced vital capacity (FVC) and forced expiratory volume in 1 s (FEV_1_) are measured to screen for pulmonary obstruction (FEV_1_/FVC ratio below the lower limit of normal). *Z*-scores are calculated based on reference equations published by Quanjer et al. [68]. Spirometry data are used to characterize the patient cohort.

### Participant timeline {13}

A participant timeline including study visits and assessments is provided in Fig. 1. Study visits take place at the University of Zurich. In addition, we offer home visits for people who are unable to visit the study center due to their health condition. The study team considers a home visit when the distance from our institute to the patient’s home is no longer than 90 min (one way).

### Sample size {14}

Sample size calculations were based on data from a population-based prospective cohort study (Zurich SARS-CoV-2 Cohort) [3]. In this cohort (*n* = 1543), 208 (13.5%) study participants reported at least one symptom 6 months after the polymerase chain reaction (PCR) diagnosed SARS-CoV-2 infection [3]. The mean (SD) health status assessed with the EQ-VAS was 73.4 ± 16.5 on a 0–100 scale. Using the mean ± SD EQ-VAS data from this cohort and considering the MID of the EQ-VAS of 8 units [31] as reference, with 80% power, the estimated sample size is 136 subjects (i.e., 68 per group). This sample size allows us to detect a difference of 8 units in the EQ-VAS between the verum and placebo groups at 12 weeks. Assuming a drop-out rate of 10%, we aim to include a total of 150 subjects.

### Recruitment {15}

We are using various recruitment channels such as the ALTEA network (https://altea-network.com/en), the Long COVID Citizen Science Board [69], and the Long COVID Network Switzerland (https://long-covid-info.ch/). In addition, we share online flyers including information about the purpose of the study, the target group, and contact details of the study team with different stakeholders.

## Assignment of interventions: allocation

### Sequence generation {16a}

We used six different groups (A, B, C, D, E, F) with three representing placebo and three representing Pycnogenol®, where the allocation was unknown to the study team (see below). A senior biostatistician, not involved in the trial, generated a list of random numbers (block sizes of six representing the six different groups) using the package *blockrand* with the software R [70]. Note that we did not use varying block sizes because the probability that the study team could guess the allocation sequence is very low and because a block size of 12 seemed to be too large. Randomization is stratified for duration of PCC-related symptoms (≤ 6 months versus > 6 months) and presence of symptomatic chronic disease(s).

### Concealment mechanism {16b}

Computer-based randomization is done within the REDCap (Research Electronic Data Capture, Vanderbilt University, USA) database [71]. Study personnel performing randomization are unable to foresee the randomization sequence in REDCap due to restriction of user rights (i.e., no access to randomization module). At the end of the baseline visit (V2), following evaluation of inclusion and exclusion criteria, study participants are randomly assigned in a 1:1 ratio to receive Pycnogenol® or placebo.

### Implementation {16c}

A senior biostatistician, not involved in the trial, generated a list of random numbers. Upon evaluation of inclusion and exclusion criteria, trained study staff allocates participants to either the Pycnogenol® or placebo group in REDCap. The database provides a code (A, B, C, D, E, F), and study staff requests the appropriate study products according to pre-defined product labels. Each of the codes represents either Pycnogenol® or placebo and remains unknown to the entire study team until the study is completed. A team from our institute, not involved in the trial, was responsible for product labeling.

## Assignment of interventions: blinding

### Who will be blinded {17a}

Trial participants, team of investigators, outcome assessors, and data analysts are blinded to minimize information and measurement bias. Investigational products are identical in appearance and appropriately labeled, and the de-identification code is only accessible to the principal investigator.

### Procedure for unblinding if needed {17b}

In the event of a medical emergency or a suspected unexpected serious adverse reaction (SUSAR), unblinding is permissible. The unblinding process of the sponsor for the respected trial participant will take place at the latest possible time before the SUSAR reporting. If unblinding is needed, the principal investigator contacts the product labeling team to initiate the unblinding procedure for the respective participant. Unblinding will be recorded in the participants electronic case report form (eCRF).

## Data collection and management

### Plans for assessment and collection of outcomes {18a}

Study personnel underwent comprehensive training of all study processes including outcome assessments and database entries. Standard operating procedures (SOPs) are available for all study-specific assessments and processes. Database entries are monitored by an internal designated team member and an external monitor. Data from study visits are collected on case report forms (CRF) and are entered directly into the database after each visit. Questionnaires, most of which are validated instruments, are completed online by study participants, or on paper, if needed. Questionnaire data are inspected before each study visit. Missing data are added, and implausible entries are verified together with the participant.

Results from standard laboratory tests (ANALYTICA Medizinische Laboratorien AG, Zurich, Switzerland) are transmitted electronically via secured e-mail and entered into eCRFs. Data from biological samples, analyzed by our internal laboratory, are entered into eCRFs by trained study personnel. At the baseline visit (V2), participants are provided with a symptom diary, which they are required to return at their final study visit (V4). The symptom diary is discussed with participants during study visits and telephone calls to minimize missing data. Study personnel enters information from the diary into the eCRFs.

### Plans to promote participant retention and complete follow-up {18b}

To promote participant retention and complete follow-up data, we planned a short study visit 6 weeks after randomization and two phone calls to stay in close contact with study participants to build trust and confidence. If a participant chooses to discontinue their involvement in the study, we follow the following 3-step process: (1) we encourage the participant to complete the follow-up assessments according to the study protocol; (2) if the participant is unwilling to complete the assessments as outlined in the protocol, we inquire if they would be willing to complete the study assessments at the time of discontinuation; (3) in the event that the participant declines to complete a study visit, we encourage them to complete the assessment for the primary endpoint as per the protocol. Finally, we offer home visits for those who are not able to attend the study center. This enables individuals with severe PCC to participate in the study, thereby enhancing the likelihood of higher retention rates in the trial.

### Data management {19}

Data is collected using REDCap [71], a secure web-based application for managing research studies hosted at the Epidemiology, Biostatistics and Prevention Institute (EBPI) at the University of Zurich, Switzerland. A unique identifier is automatically generated within REDCap and is used for each participant on all study documents. All data are stored on a password-protected server and regular backups and software updates are performed. Team members have password-protected accounts with different levels of user access rights based on their roles in the study. Access to the database requests two-factor authentication.

Study participants complete online surveys upon e-mail invitation. Range checks are implemented in the database including online surveys to minimize data entry errors.

Our internal data quality manager checks database entries for correctness and plausibility. An external data monitor (MEDICRO GmbH, Petersaurach, Germany) ensures that the conduct, documentation, and reporting are in accordance with the study protocol, defined SOPs, Good Clinical Practice (GCP), and legal regulations. This includes correct issuance of investigational products as per randomization in REDCap and evaluation of the completeness of primary outcome information as well as completeness and correctness of adverse event reporting for all participants. Additionally, inclusion/exclusion criteria are checked for 50% of randomly selected participants. The data monitor is tasked with conducting various monitoring visits using a mix of on-site and off-site monitoring activities, including written reporting. The following visits and responsibilities are covered and carried out: pre-study visit, study initiation visit, routine monitoring visits, and close-out visit.

Key data to be monitored as per contract:

- 100% source data verification for the first 5 study participants
- 100% source data verification for an additional 30 randomly selected subjects (20% of *N* total)
- 100% completion of case report forms including subject existence (no medical records)
- 100% investigational medicinal product (IMP) dispensation
- 100% primary endpoint assessment (EQ-VAS)
- 50% inclusion/exclusion criteria verification for randomly selected participants
- 100% adverse events (AE)/SAE reporting
- Written reporting and review

Upon database closure, all data will be stored on servers of the University of Zurich, accessible to REDCap administrators. Access to study data and files is restricted to authorized study personnel. Coded data will be stored for 10 years. Paper documents that are generated in the context of the study are stored in a locked study closet. Prior to data analysis, data exports will be checked for outliers or implausible values.

### Confidentiality {27}

Confidentiality is ensured by utilizing participant identification codes to correspond to medical information and treatment data in the computer files. Each study participant receives a unique identifier that is automatically generated within the REDCap database. This code will be used for all study documents, labeling of blood tubes, and investigational products. The information linking the identifying personal data with the unique participant code is stored separately on a password-protected participant identification list on a secure server of the University of Zurich. After finalization of data collection, the password of the participant identification list will be changed so that the file will remain accessible only to the sponsor and principal investigator (PI).

### Plans for collection, laboratory evaluation and storage of biological specimens for genetic or molecular analysis in this trial/future use {33}

Standard clinical parameters are analyzed externally by a certified diagnostic laboratory (ANALYTICA Medizinische Laboratorien AG, Zurich, Switzerland). Study-specific analyses of blood biomarkers are done in our in-house laboratory. Plasma samples for the in-house analyses are aliquoted and stored frozen until sufficient number of samples are acquired for batch analysis.

If consent for further use of data and biological material in coded form has been given, surplus samples after completion of the PYCNOVID study will be transferred to our biobank, where they will be preserved for potential future research use. In case that the biological material is no longer needed or consent for further use has not been given or is withdrawn with the request to destroy the remaining samples, it will be destroyed in compliance with the University of Zurich standards for biological waste disposal and will be documented in a project-specific laboratory journal.

## Statistical methods

### Statistical methods for primary and secondary outcomes {20a}

#### Primary outcome

The primary analysis will be done following the intention-to-treat (ITT) principle. All randomized participants having received at least one dose of Pycnogenol® will be included in this analysis. We will use multivariable linear regression models to compare EQ-VAS values at 12 weeks adjusted for baseline values and stratification factors, i.e., PCC duration (≤ 6 months versus > 6 months) and presence of any chronic disease(s).

#### Secondary outcomes

Secondary endpoints will be analyzed using linear regression models adjusting for baseline values and stratification factors. Changes in symptoms over time for dyspnea, fatigue, cognitive impairment (“brain fog”), and post-exertional malaise will be analyzed with McNemar’s test. Changes in symptom severity over time will be analyzed descriptively. A per protocol analysis is done for all secondary outcomes similar to the one described for the primary outcome.

#### Other outcomes

Using baseline data, we aim to investigate associations between objectively measured physical activity, symptom burden and severity, functional exercise capacity, and quality of life. Descriptive statistics and regression models will be applied.

### Interim analyses {21b}

Interim analyses are not planned.

### Methods for additional analyses (e.g., subgroup analyses) {20b}

We will conduct a per-protocol analysis comparing adherent versus less-adherent participants. Adherence is assessed by calculating the percentage of taken/unused study products over the course of the study. We will compare groups of participants who took ≤ 70% of capsules versus those who took > 70% of capsules during the study to investigate any potential effect of adherence on primary and secondary outcomes.

### Methods in analysis to handle protocol non-adherence and any statistical methods to handle missing data {20c}

Substantial deviations from the original statistical plan will only be done if necessary due to unforeseen circumstances. Any such deviation will be reported and justified in the final report and the resulting manuscript(s). Missing follow-up data of study participants who did not conduct assessments at the final study visit (V4) will be imputed using multiple imputation by chained equations. The results from this procedure will be compared to the ones from the complete case analysis.

### Plans to give access to the full protocol, participant-level data and statistical code {31c}

We plan to deposit an anonymized dataset within available repositories (e.g., Dryad, Zenodo) to facilitate open access for other researchers. The study consent form will explicitly inform participants about the reuse and sharing of anonymized data. Identifiable information will not be shared with anyone outside of the research team. The statistical code will be made available in the publications.

## Oversight and monitoring

### Composition of the coordinating center and trial steering committee {5d}

The study team is composed of physicians and medical trainees, epidemiologists and senior researchers, certified study nurses, and a doctoral student with a background in biomedicine and administrative staff. Senior staff members have extensive experience in clinical trial conduct and bear the responsibility of supervising and training care providers and outcome assessors. Prior to the start of the study, a comprehensive training program was established, and pilot testing of study procedures and measurements was done. Core team members including the study manager and PI meet on a weekly basis to discuss day-to-day questions in regard to study conduct, study progress, and recruitment goals. The sponsor receives regular updates about meeting outcomes. Meetings with the sponsor are done on a monthly basis. The REDCap database manager and internal study monitor meet regularly to ensure the maintenance of high-quality data. An external data monitor visits the study site several times throughout the study conduct.

### Composition of the data monitoring committee, its role and reporting structure {21a}

A data monitoring committee is not needed for this clinical trial since the investigational product Pycnogenol® is already approved by the Swiss Authority for Pharmaceutical Products (Swissmedic) and is available over the counter. The approved market product (Swissmedic number 49969 and 57716) is available as 20 mg tablet. In this study, we use 50 mg capsules.

### Adverse event reporting and harms {22}

The risk for the participants is considered minimal as the investigational product Pycnogenol® is generally well tolerated and has been proven to be save in multiple clinical trials before [20, 21, 29]. All adverse events occurring after the baseline visit (V2) are collected in the eCRFs in REDCap. Once an adverse event is detected, it will be followed up until its resolution or until it is judged to be permanent. A SAE is classified as any untoward medical occurrence that (i) results in death, (ii) is life-threatening, (iii) requires in-patient hospitalization or prolongation of existing hospitalization, (iv) results in persistent or significant disability/incapacity, or (v) is a congenital anomaly/birth defect. In addition, important medical events that may not be immediately life-threatening or result in death, or require hospitalization, but may jeopardize the participant or may require intervention to prevent one of the outcomes listed above are usually considered serious. SAEs are followed up until resolution or stabilization. Participants with ongoing SAEs at study termination will be further followed up until recovery or until stabilization of the health threatening condition. SAEs will be reported immediately and within a maximum of 24 h to the sponsor, who will re-evaluate the SAE and return the form to the site. SAEs resulting in death are reported to the Competent Ethics Committee via Business Administration System for Ethical Committees within 7 days.

### Frequency and plans for auditing trial conduct {23}

A quality assurance audit/inspection may be conducted by the competent authority (Swiss Agency for Therapeutic Products) or the competent ethical committee, respectively. The auditors/inspectors will have access to all source data/documents such as eCRFs, written informed consent, laboratory and medical test results, the investigator’s study-related files and correspondence, and documentation that is relevant to this trial. All involved parties will keep the participant data strictly confidential.

### Plans for communicating important protocol amendments to relevant parties (e.g., trial participants, ethical committees) {25}

Substantial amendments can only be implemented after approval of the competent ethical committee and involved competent authority (Swissmedic), respectively. All non-substantial amendments are communicated to the competent ethical committee within the annual safety report and as soon as possible to the involved competent authority, if applicable.

Under emergency circumstances, necessary deviations from the protocol may proceed without prior approval of the sponsor and the competent ethical committee/competent authority to protect the rights, safety, and well-being of human subjects. Such deviations will be documented and reported to the sponsor and the competent ethical committee/competent authority as soon as possible.

### Dissemination plans {31a}

After the last study visit (V4), participants receive the results from their diagnostic blood tests, electrocardiogram, spirometry, and MoCA test in written form.

Results of this trial will be published in peer-reviewed international scientific journals. Furthermore, summary reports presenting the key findings of the study will be disseminated to various stakeholders including those affected by PCC in a clear and accessible language. Those include, among others, the ALTEA network (https://altea-network.com/en), the Long COVID Citizen Science Board [69], the Long COVID Network Switzerland (https://long-covid-info.ch/), as well as the LinkedIn account and the communication platform ges.UND? (https://www.uzh.ch/blog/ebpi-gesund/) of our institution.

## Discussion

This 12-week single-center, placebo-controlled trial investigates the effect of Pycnogenol® on patient-reported health status in people affected by PCC. Our study team has significant experience working with people affected by PCC [1, 3, 11]. The instruments and assessment methods used in this study are well established at our institution and have been used by our team in several research projects including RCTs. We have involved people living with PCC in the study development process and acquired feedback for the primary endpoint selection and discussed options to optimize the study visits for people suffering from PCC. Post-exertional malaise is a frequently reported symptom in PCC [1, 3], and our visits (including travel) may pose a potential burden to participants. We perform all measurements in a quiet environment and allow participants to rest anytime during the study visits to minimize the risk for worsening of symptoms (“crashes”). We specifically assess possible worsening of symptoms following each study visit and offer to skip the exercise test in the next visit, for example, to keep testing burden as low as possible.

Recruiting volunteers affected by a chronic condition for trials always presents a challenge. During the SARS-CoV-2 pandemic, we have established a well-working network composed of professionals and those affected by PCC including the development of a Long COVID Citizen Science Board [69]. This network will facilitate the successful recruitment of participants for this study. Additionally, we offer home visits for those who are unable to visit the study center due to their health condition. All necessary infrastructure including a bus equipped with a fridge allowing transport and storage of biological samples is available. Home visits not only facilitate the enrolment of individuals with severe PCC but also enhance the generalizability of our study findings to encompass a broader spectrum of disease severity.

We have undertaken all efforts to design a methodologically robust trial, including blinding of study participants and the entire study team (investigators, sponsor, outcome assessors, data analyst) to reduce bias. Computer-based randomization and proper concealment of random allocation minimizes confounding and selection bias. We use validated instruments and integrated core outcome measures for studying the efficacy of Pycnogenol® versus placebo on PCC-related sequelae [72]. The primary endpoint was established with people with lived experience, and they confirmed that the EQ-VAS captures their daily symptom burden well. Due to the episodic nature of PCC-related symptoms and their impact on daily life, we assess health status (EQ-VAS) daily over 7 days at baseline and post intervention to be able to best capture those fluctuations.

The clinical diagnosis of PCC is complex and challenging [73]. Not all individuals enrolled in this study will have a physician diagnosis of PCC, which may introduce some uncertainty. However, we request confirmation of a SARS-CoV-2 infection by a positive PCR or rapid antigen test for professional use or a written medical report including a physician diagnosis of PCC. Additionally, we evaluate the plausibility of PCC-related symptoms by self-reported symptoms, symptom duration and start of persisting symptoms prior to inclusion into the study.

## Trial status

The study protocol version 6.0, 13.10.2023, was approved by the Competent Ethics Committee of the Canton of Zurich, Switzerland. The recruitment started on the 7 June 2023 and will be finished by 1 July 2024 (estimated).

## Data Availability

The final dataset will be stored and maintained at the Epidemiology, Biostatistics and Prevention Institute (EBPI) at the University of Zurich. We intent to make all publications available as open access. Data request proposals will be overseen by the core study team (AA, JSF, MAP, MR, TR). Their final decision about data sharing will be binding. Possible data transfers will need to comply with the data transfer agreement guidelines.

## Abbreviations

AE: Adverse event
COVID-19: Coronavirus disease
CRQ: Chronic Respiratory Questionnaire
CRP: C-reactive protein
eCRF: Electronic case report form
ECG: Electrocardiogram
EQ-5D-5L: EuroQol Questionnaire (five-level quality of life instrument)
EQ-VAS: EuroQol Visual Analogue Scale
FACIT-F: Functional Assessment of Chronic Illness Therapy—Fatigue Scale
GCP: Good Clinical Practice
ITT: Intention to treat
MID: Minimal important difference
MoCA: Montreal Cognitive Assessment
PCC: Post-COVID-19 condition
PCR: Polymerase chain reaction
REDCap: Research Electronic Data Capture, Vanderbilt University, USA
RCT: Randomized controlled trial
SAE: Serious adverse event
SARS-CoV-2: Severe acute respiratory syndrome coronavirus type 2
SUSAR: Suspected unexpected serious adverse reaction
